# Age-associated stresses induce an anti-inflammatory senescent phenotype in endothelial cells

**DOI:** 10.18632/aging.100622

**Published:** 2013-12-12

**Authors:** Paul R. Coleman, Garry Chang, Gabor Hutas, Matthew Grimshaw, Mathew A. Vadas, Jennifer R. Gamble

**Affiliations:** Centre for the Endothelium, Vascular Biology Program, Centenary Institute, Locked Bag #6, Newtown NSW, Australia and the University of Sydney, NSW, Australia

**Keywords:** senescence, endothelial cells, inflammation, ARHGAP18, SENEX, ageing

## Abstract

Age is the greatest risk factor for cardiovascular disease. In addition, inflammation and age (senescence) have been linked at both the clinical and molecular levels. In general, senescent cells have been described as pro-inflammatory based on their senescence associated secretory phenotype (SASP). However, we have previously shown that senescence induced by overexpression of *SENEX (or ARHGAP18)*, in endothelial cells results in an anti-inflammatory phenotype. We have investigated, at the individual cellular level, the senescent phenotype of endothelial cells following three of the chief signals associated with ageing; oxidative stress, disturbed flow and hypoxia. All three stimuli induce senescence and, based on neutrophil adhesion and expression of the adhesion molecules E-selectin and VCAM-1, a population of senescent cells is seen that is resistant to inflammatory stimuli and thus we define as anti-inflammatory. The proportion of anti-inflammatory cells increases with time but remains stable at approximately 50% by eight days after induction of senescence, suggesting that these are stable phenotypes of endothelial cell senescence. Similar to other senescent cell types, p38MAPK blockade inhibits the development of the pro-inflammatory phenotype but unique to EC, there is a corresponding increase in the number of anti-inflammatory senescent cells. Thus stress-induced senescent endothelial cells display a mosaic of inflammatory phenotypes. The anti-inflammatory population suggests that senescent endothelial cells may have an unique protective role, to inhibit uncontrolled proliferation and to limit the local inflammatory response.

## INTRODUCTION

Endothelial cell senescence is linked to both aging and vascular pathologies. With age there is an increase in the markers of senescence in the vasculature, including senescence associated β-galactosidase (SA β-gal) and p16. Increased numbers of senescent endothelial cells (ECs) are found in atherosclerotic plaques of human aorta and coronary arteries [1], in coronary vessels of patients with ischaemic heart disease [2], in vessels from diabetic rats [3] and EPC from diabetics undergo heightened senescence postulated to be one of the causes of the impaired neovascularisation seen in diabetes [4]. Furthermore, many stimuli linked to cardiovascular disease (CVD) induce senescence, for example, asymmetrical dimethylarginine, and homocysteine [5], and reactive oxygen species (ROS) [6]. Senescent ECs have also been identified in the tumor vasculature in glioma [7].

The three major stress factors associated with age are oxidative stress, shear stress and hypoxia. Oxidative stress results from the generation of ROS and vascular aging is associated with an increase in ROS [8] and these species, which include superoxide anion radicals and hydrogen peroxide (H_2_O_2_) are found in all layers of the diseased arterial wall. EC at the sites of bifurcations and branching are subjected to increased and chronic changes in shear forces [9]. At these points there is increased EC turnover likely as a consequence of the activation through the changes in shear stress and is likely a mechanism to maintain vascular integrity following cell apoptosis. These sites are also the atherosclerotic prone regions of vessels [10]. Finally, hypoxia is a major driver of angiogenesis in tumours and inflammation and also is seen in vascular diseases such as diabetes and atherosclerosis[11-13].

To date, senescent ECs have been reported to display several proteins that are involved in the pro-inflammatory, pro-thrombotic phenotype of the endothelium in human atherosclerosis. These include interleukin-1α, the intercellular adhesion molecule ICAM-1 [14] and plasminogen activator inhibitor-1 [15]. Furthermore, both nitric oxide (NO) production and endothelial nitric oxide synthase activity are reduced in senescent EC [16]. The production of ROS is also increased in senescent cells leading to a further decrease in the bioavailability of NO [17] suggesting a pro-inflammatory state. However, somewhat surprisingly, our recent work [18] showed that senescent EC induced by the overexpression of the gene *SENEX (ARHGAP18)* induces an anti-inflammatory phenotype. Thus we postulated that this type of senescence could act to limit or prevent the development of disease. Given this seemingly contradictory result to that of previous studies in the senescence field, the aim of our present work was to dissect, at the cellular level, the response of EC to age-associated stress. Our results show that three of the major stresses to the vasculature and which are increased in ageing, namely oxidative stress, disturbed shear stress and sustained hypoxia all induce EC senescence. Further, a population of senescent ECs is refractory to stimulation by TNFα and displays an anti-inflammatory phenotype, while other senescent cells respond like normal EC to TNFα stimulation and become pro-inflammatory. The mosaic of inflammatory senescent cells is constant overextended time suggesting that these are stable and end stage phenotypes. Aged individuals show a general loss of immune function, which has been mainly attributed to a decline in T cell function, termed immune-senescence and the increase in suppressive factors secreted by macrophages [19, 20]. However, our results would suggest the endothelium is also important in this decline in immune function, given its central role in the extent and duration of an inflammatory response. Thus senescence in the vasculature may play a dynamic role, to promote or restrain the inflammatory response.

## RESULTS

### Oxidative, flow and hypoxic stress induces senescence

**Figure 1:**
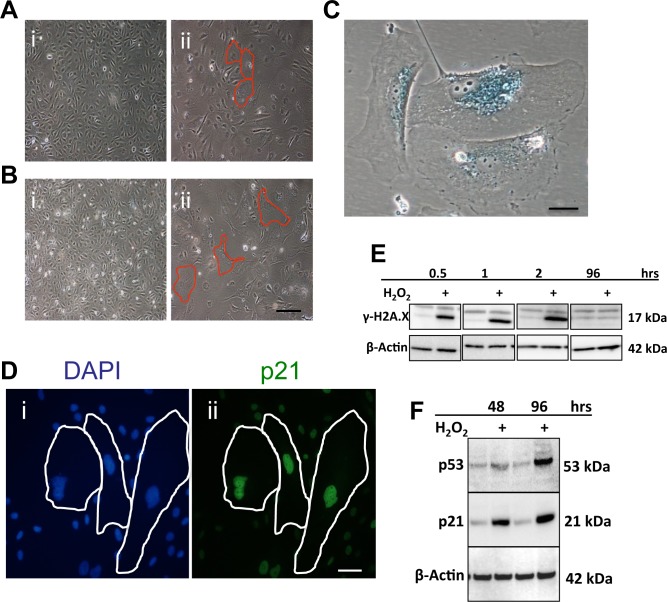
Oxidative stress induces senescence in ECs (**A**) HUVECs were treated with 0.2mM H_2_O_2_. After 2 days cells began to demonstrate the morphology of EC senescence (ii-Red outline) compared to untreated cells (i). (**B**) After a further 2 days the number of senescent cells had increased and the size of the senescent cells had also increased (ii-Red outline), compared to untreated cells (i). This is a representative of 10 HUVEC lines. Bar=220μm. (**C**) HUVECs were treated with 0.2 mM H_2_O_2_ and after 4 days stained for SA-β-gal. This is a representative of 10 HUVEC lines. Bar=25μm. (**D**) HUVECs were untreated (i) or treated with 0.2 mM H_2_O_2_ and after 4 days stained using immunofluorescence for DAPI (i) and p21 (ii). This is a representative of 5 HUVEC lines. Bar=50μM. (**E**) and (**F**) Cells were treated with 0.2 mM H_2_O_2_ and lysates analysed for levels of γH2A.X (**E**), p53 and p21 (**F**). β-Actin was used as a loading control. This is a representative of 8 HUVEC lines.

We tested the senescence response of EC to the three stress situations associated with age and EC dysfunction Sub-lethal doses of H_2_O_2_ as a method to induce oxidative stress, results in robust senescence in human umbilical vein endothelial cells (HUVECs). Within 48 hours of treatment with 0.2mM of H_2_O_2_ the cells began to demonstrate a flattened and enlarged senescent morphology and by 4 days the cells had further increased in size (Figure 1A and B respectively). Senescence was confirmed by the large cells being positive for SA-β-Gal (Figure 1C), and p21 (Figure 1D). The DNA damage response (DDR) impacting on p53/p21 is a key pathway in senescence induction and previous studies have shown H_2_O_2_ treatment activates the DDR. The DDR response, as identified by the marker γH2A.X, was upregulated within 30mins of H_2_O_2_ treatment (Figure 1E) and remained elevated for approximately the next 2 hours, returning to baseline thereafter. Consistent with this we found an upregulation of p53 and p21 protein levels, 48 and 96 hrs following H_2_O_2_ treatment, at a time when the senescent phenotype is detected (Figure 1F).

**Figure 2:**
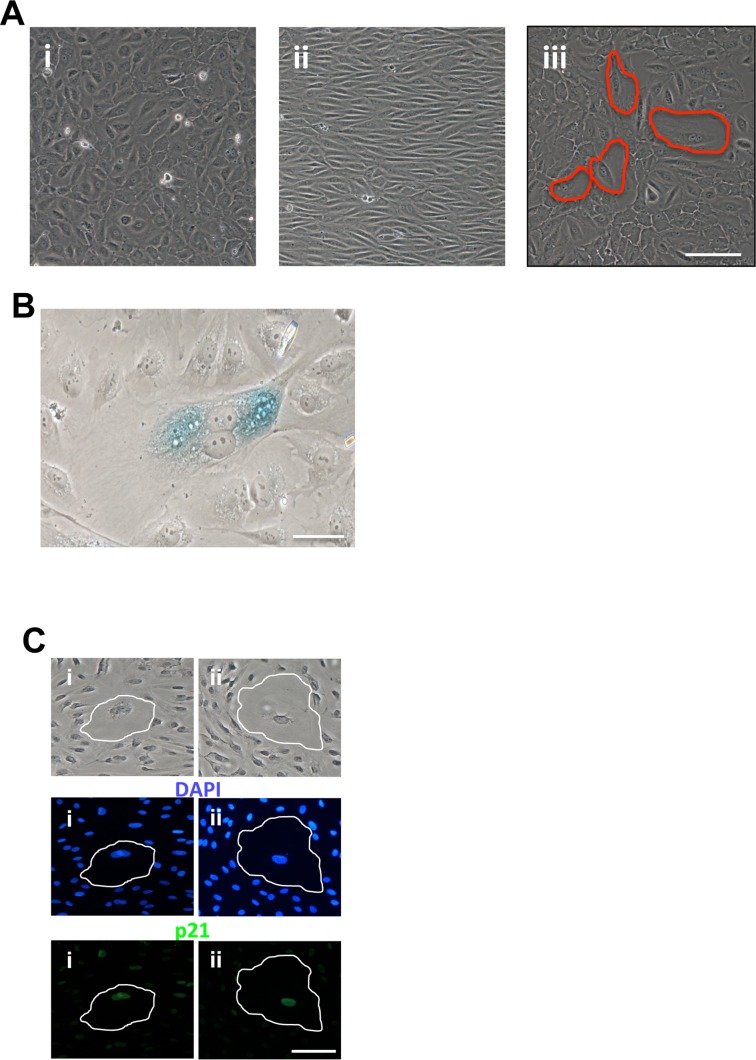
Induction of EC senescence with disturbed flow (**A**) HUVECs were left in static conditions (i) or subjected to 48hrs of flow at 20dyne/cm^2^ (ii) or 2dyne/cm^2^ (iii). Senescent cells are circled in red. This is a representative of 5 HUVEC lines. Bar=100μm (**B**) Cells exposed to 2dyne/cm^2^ flow for 48hrs were fixed and stained for SA-β-gal. This is a representative of 3 HUVEC lines. Bar=50μM. (**C**) Cells exposed to 2dyne/cm^2^ flow for 48hrs, fixed and stained for DAPI (blue) and p21 (green). Two representative senescent cells have been highlighted. This is a representative of 3 HUVEC lines. Bar=100μm.

Senescence was also seen under disturbed flow. Cells placed under static conditions showed normal cobblestone morphology (Figure 2Ai). Under laminar flow conditions at 20dynes/sec, they began to elongate and align after 48 hours but no senescent cells were visible (Figure 2Aii). However, cells subjected to low shear stress at 2dynes/sec for 48 hours showed a significant number of these large flattened cells (Figure 2Aiii). These were confirmed to be senescent by this morphology being highly vacuolated and flattened often polyploidy, staining positive for SA-β-Gal (Figure 2B) and for p21 (Figure 2C).

**Figure 3:**
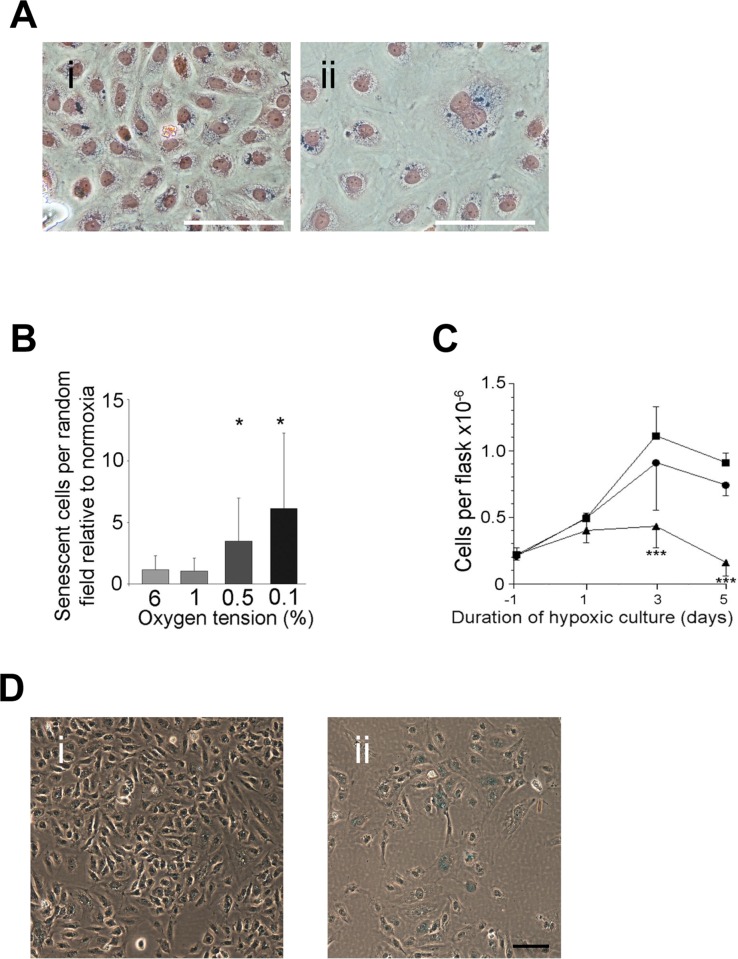
Hypoxia induced senescence (**A**) HUVECs were cultured in normoxic conditions (i) or under hypoxic conditions (0.5% oxygen) for 5 days and then stained for SA-β-gal and Eosin counter-staining (ii). Bar=100μm. (**B**) The number of senescent cells per random field was counted after 5 days treatment at different oxygen tensions. The number of senescent cells at each oxygen tension is normalised to the number of cells in normoxic conditions using matched lines (mean of four experiments per oxygen tension ± SD. * P<0.05, paired Student's t-test). (**C**) Cell growth was compared at normoxia (squares), 1% oxygen (circles) and 0.5% oxygen (triangle) for 5 days. Cells were seeded 24 hours (Day −1) prior to being placed into the hypoxic incubator on day 0 (mean of four experiments per oxygen tension ± SD. *** P<0.001, paired Student's t-test). (**D**) HUVECs were untreated (i) or treated with 10μM DFO for 72 hrs (ii) then fixed and stained for SA-β-gal. This is a representative of 3 HUVEC lines. Bar=100μm.

Severe and prolonged hypoxia also induced senescence as judged by SA-β-Gal positivity, polyploidy, large flatten appearance and the increase in vacuoles (Figure 3A). Senescent cells were induced after 5 days with at least 0.5% hypoxia (Figure 3B). 1% hypoxia showed little if any senescence (Figure 3B). Consistent with this senescence induction there was a decrease in cell numbers over the 5 days (Figure 3C). Similar senescence induction was seen when EC were treated with deferoxamine (DFO), a drug that inhibits prolyl hydroxylase and stabilises HIF1a and HIF2a [21] (Figure 3D).

Together these results show that senescence is induced under the age-related stress conditions of oxidative stress, disturbed flow and hypoxia.

### Inflammatory phenotype of senescent cells

**Figure 4:**
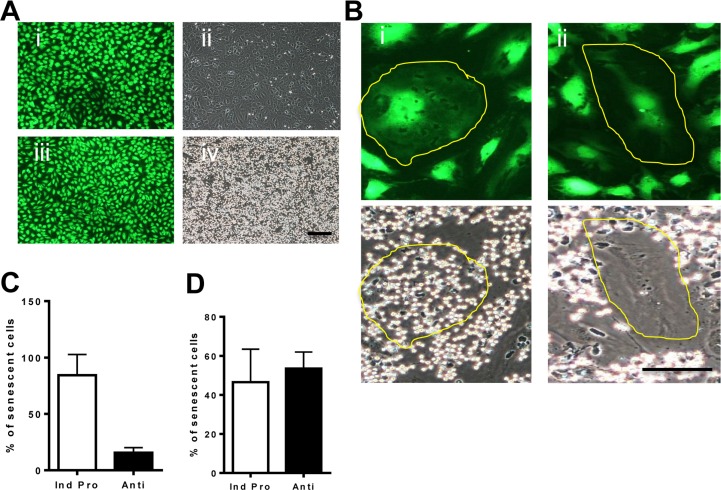
Oxidative stress induced senescence has a unique inflammatory phenotype (**A**) Normal HUVECs were stained with cell tracker green for visualization (i and iii) and then neutrophil adhesion was determined by a static adhesion assay. Cells were either untreated (i,ii) or treated with 5ng/ml of TNFα for 5 hours (iii, iv). (**B**) HUVECs were treated with 0.2 mM H_2_O_2._ After 4 days the cells were stimulated with 5ng/ml TNFα and stained with cell tracker green for visualization. Senescent cells are highlighted with a yellow line. Representative photos of the pro-inflammatory senescent cells (i) and the anti-inflammatory senescent cells (ii) are shown. This is a representative of 6 HUVEC lines. Bar=25μm. (**C**) From the photographs taken in (**B**) the number of anti- and pro-inflammatory senescent cells was determined and given as the % of total senescent cells. This is the representative of the mean +/− SD of 200 senescent cells from 6 HUVEC lines. (**D**) From videos taken of neutrophil rolling and adhesion (Videos 1-5) the percentage of pro and anti-inflammatory senescent cells after TNFα stimulation was determined. This is a representative of the mean +/− SD of 220 senescent cells from 4 HUVEC lines.

The senescence phenotype induced by oxidative stress was probed in more detail, at the individual cellular level, using neutrophil adhesion as a measure of EC inflammatory status. EC were treated with H_2_O_2_ for 4 days. Normal EC (stained with cell tracker) failed to support any neutrophil adhesion in the absence of TNFα stimulations (Figure 4Ai and ii respectively). Following TNFα stimulation, normal EC supported neutrophil adhesion as we have previously described [22] (Figure 4Aiii and iv). Following TNFα stimulation the H_2_O_2_-induced senescent cells displayed two populations of cells. One population of senescent EC responded to the stimulation similar to normal non-senescent cells and supported neutrophil adhesion. These were classified as pro-inflammatory senescent cells (Figure 4Bi). The second population of senescent cells did not support neutrophil adhesion and these were termed anti-inflammatory (Figure 4Bii). Four days after H_2_O_2_ stimulation, we found approximately 80% of the senescent cells were pro-inflammatory under the conditions of these static assays and the remainder displayed the anti-inflammatory phenotype (Figure 4C).

To determine whether these differing phenotypes were also seen in more physiological conditions, we assessed the senescence phenotypes with neutrophil adhesion performed under flow conditions. No neutrophil rolling or adhesion was seen in normal EC in the absence of TNFα (Video1), although neutrophil rolling and adhesion was seen after TNFα stimulation (Video2). In the H_2_O_2_-induced senescent cells even after TNFα stimulation some senescent cells failed to support neutrophil adhesion and at this velocity, the neutrophils skipped across the senescent EC (Video3-cell is circled in red). Other senescent cells after TNFα stimulation showed neutrophil adhesion (Video4-cell is circled in red) similar to normal activated EC. When the phenotype of the different TNFα-stimulated senescent cells was calculated, the anti-inflammatory population was approximately 50% of the total number of senescent cells (Figure 4D). This would suggest that under static conditions, some senescent cells have sufficient adhesive molecules to maintain the adhesion but that under flow conditions the interactions are weak and rolling and adhesion cannot be maintained.

### Senescent cells have altered adhesion molecule expression

**Figure 5:**
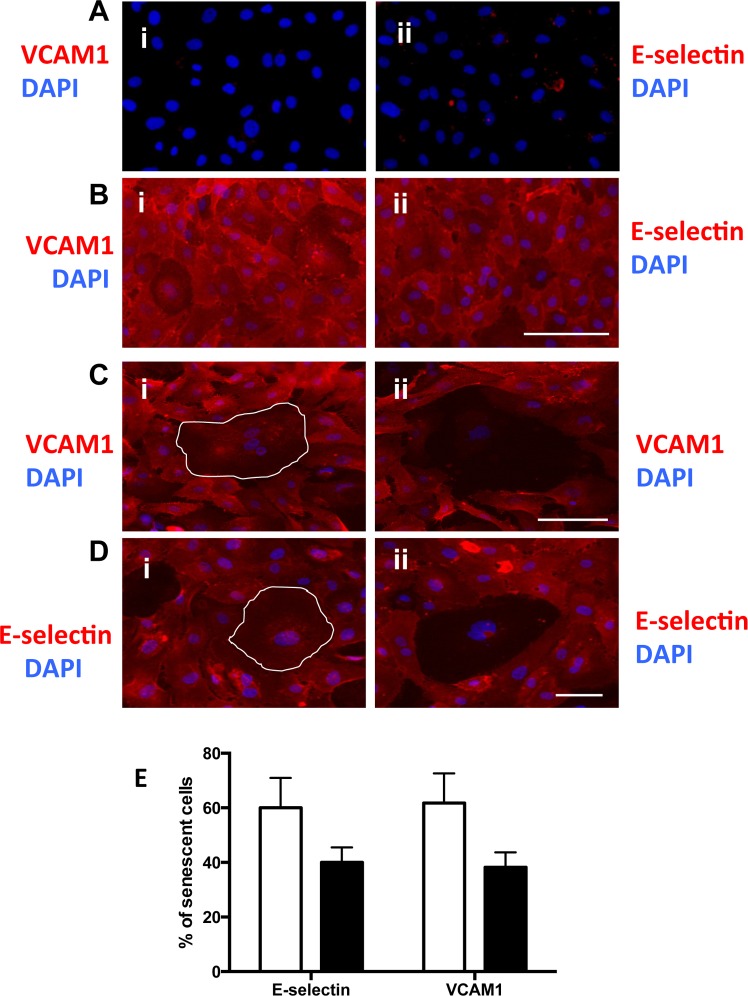
Senescent cells have altered adhesion molecule expression (**A** and **B**) HUVECs were either untreated (**A**) or stimulated with 5ng/ml TNFα (**B**) and stained for the surface expression of VCAM-1 (red) (i) and E-selectin (red) (ii) and co-stained with DAPI (blue). This is a representative of 6 HUVEC lines. Bar=100μm. (**C**) HUVECs were treated with 0.2 mM H_2_O_2_, for 4 days, stimulated with 5ng/ml TNFα then fixed and stained for the surface expression of VCAM-1 (red) and co-stained with DAPI (blue). Representative pro-inflammatory senescent cells (i) and anti-inflammatory senescent cells (ii) are shown, highlighted by the white outline. This is a representative of 6 HUVEC lines. Bar=100 μm. (**D**) Cells were treated as in (**C**) but stained for the surface expression of E-selectin (red) and co-stained with DAPI (blue). This is a representative of 6 HUVEC lines. Bar=100μm. (**E**) From the photographs taken in (**C** and **D**) the number of anti and pro inflammatory senescent cells was determined. This is a representative of the mean percentage +/− SD of 150 senescent cells from 6 HUVEC lines.

The adhesion of neutrophils to ECs is controlled by adhesion molecules such as E-selectin and VCAM-1 whereas ICAM-1 and IL-8 are responsible for their transmigration [23]. We assessed the expression of E-selectin and VCAM-1 on senescent cells. Under basal unstimulated (no TNFα) conditions EC do not express any E-selectin or VCAM-1 (Fig 5Ai and ii) whereas normal EC upregulated these adhesion molecules in response to TNFα (figure 5Bi and ii). On the H_2_O_2_-induced senescent cells stimulated with TNFα, some senescent cells expressed VCAM-1 and E-selectin (Figure 5Ci and Di) while others failed to express these adhesion molecules (Figure 5Cii and Dii). When assessed 4 days after H_2_O_2_ treatment, 60% +/− 5.5 of the senescent cells showed normal levels of adhesion molecules after TNFα stimulation whereas 40+/− 5.5% of the senescent cells failed to express the proteins on their cell surface (Figure 5E).

**Figure 6:**
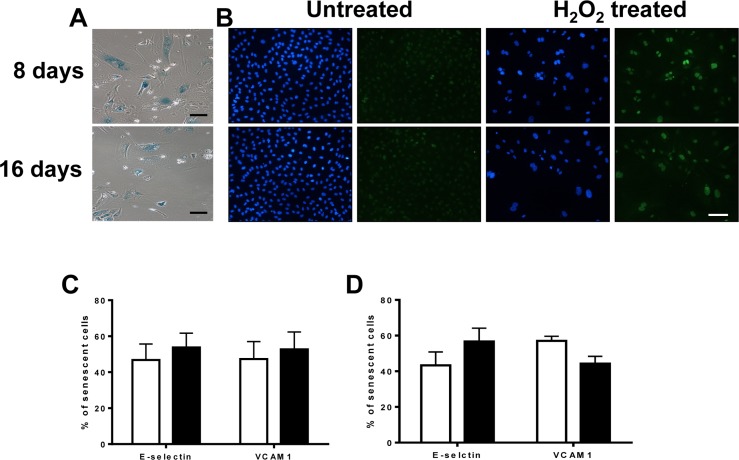
The anti-inflammatory phenotype is stable and long lasting (**A**) HUVECs were treated with 0.2mM H_2_O_2_ and after 8 or 16 days fixed and stained for SA-β-gal. This is a representative of 3 HUVEC lines. Bar=100μm. (**B**) HUVECs were treated with 0.2mM H_2_O_2_ and after 8 and 16 days fixed and stained for DAPI (blue) and p21 (green). This is a representative of 3 HUVEC lines. Bar=100μm. The percentage of induced pro and anti-inflammatory senescent cells on day 8 (**C**) and 16 (**D**) based on E-selectin and VCAM-1 expression were determined. This is a representative of the mean percentage +/− SD of 80 senescent cells from 3 HUVEC lines.

To determine whether these were 2 separate populations of senescent cells or a progression in the development of the inflammatory senescent phenotype, we assessed the inflammatory status at day 8 and day 16 after H_2_O_2_ addition. In this case the cells were stimulated with the H_2_O_2_ on Day 0 and then left for either 8 or 16 days with no further stimulation. The majority of the cells on each day were senescent as confirmed by SA-β-Gal (Figure 6A) and p21 positivity (Figure 6B). By day 8 (Figure 6C) the ratio of anti-inflammatory to pro-inflammatory senescent cells, as assessed by adhesion molecule expression, was approximately 1:1 and this was similar on day 16 (Figure 6D). These results suggest that oxidative stress induces a stable mosaic inflammatory phenotype of senescent EC, judged either by neutrophil adhesion or adhesion molecule expression, where the anti-inflammatory phenotype and the pro-inflammatory phenotype exist in concert.

**Figure 7:**
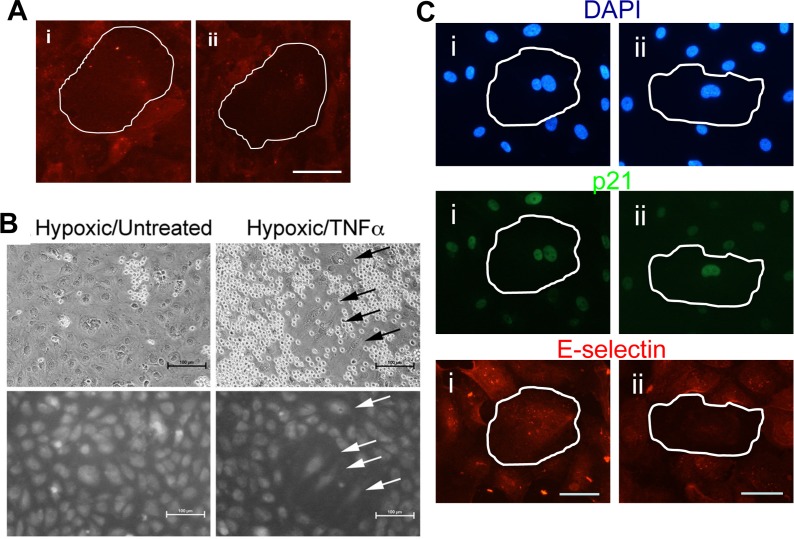
Shear stress and hypoxia induce an anti-inflammatory phenotype (**A**) HUVECs were subjected to 48hrs of flow at 2dyne/cm^2^. The cells were then treated with 5ng/ml of TNFα for 5 hours, fixed and stained for E-selectin (red). Induced pro-inflammatory senescent cells (i) and anti-inflammatory senescent cells (ii) are shown. A senescent cell is highlighted by the white outline. This is a representative of 3 HUVEC lines. Bar=100μm. (**B**) Binding of neutrophils to HUVECs that had been cultured at 0.5% oxygen for five days followed by treatment for six hours with 5ng/ml TNFα (left hand panel). Upper panels show light microscopy of neutrophils upon HUVECs. Lower panels show CMFDA-labelled HUVEC monolayers. Cells with a senescent morphology are indicated with an arrow. Hypoxic treated cells that were not stimulated with TNFα are shown in the left hand panel. Shown are representative images of three experiments using independent HUVEC lines. (**C**) HUVCs exposed to 10μM DFO for 72 hrs were treated with 5ng/ml of TNFα for 5 hours, fixed and stained for p21 (green), E-selectin (red) and co-stained with DAPI (blue). Representative pro-inflammatory senescent cells (i) and anti-inflammatory senescent cells (ii) are shown, highlighted by the white outline. This is a representative of 3 HUVEC lines. Bar=50μm.

We assessed the inflammatory phenotype of senescent cells induced in response to low shear stress and hypoxia. Senescent cells induced by low shear stress showed the anti- (Video5-cell is circled in red) and pro-inflammatory adhesive phenotype (Video6-cell is circled in red) when these neutrophil adhesion assays were performed under flow conditions. This is in contrast to the normal rolling on TNFα treated non-senescent cells (Video2). Senescent cells induced by low shear stress (p21 positive) also showed those that expressed cell surface E-selectin and those that failed to express E-selectin after TNFα stimulation (Figure 7A). The anti-inflammatory phenotype was also seen in the hypoxia-induced senescent cells as judged by the lack or reduced adhesion of neutrophils (Figure 7B) and E-selectin expression (Figure 7C).

**Figure 8:**
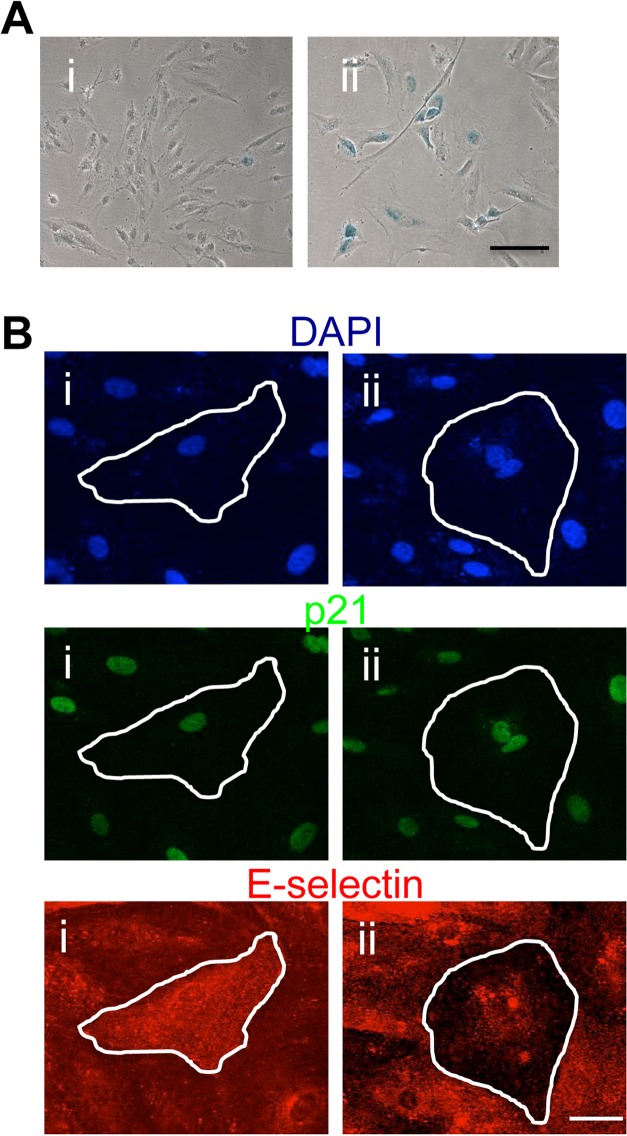
Human coronary artery EC display the anti-inflammatory phenotype (**A**) HCAEC were either untreated (i) or treated with 0.2mM H_2_O_2_ (ii)and after 4 days fixed and stained for SA-β-gal. This is a representative of 3 separate experiments. Bar=100μm. (**B**) HCAEC were treated with 0.2mM H_2_O_2_ and after 4 days stimulated with 5ng/ml of TNFα for 5 hours, fixed and stained for p21 (green), E-selectin (red) and co-stained for DAPI (blue). Representative pro-inflammatory (i) and anti-inflammatory senescent cells (ii) are shown, highlighted by the white outline. This is a representative of 3 separate experiments. Bar=100μm.

Human coronary artery EC also became senescent after H_2_O_2_ treatment, as judged by SA-β-Gal staining (Figure 8A). Further they displayed pro- and anti-inflammatory senescent cells after TNFα stimulation (Figure 8B). Therefore the anti-inflammatory phenotype is a common phenotype in different EC and not due to an enhancement of a subpopulation within the venous EC.

Taken together these results show that senescence induced by oxidative stress, disturbed flow and hypoxic conditions all generate anti-inflammatory senescent ECs. Further, this anti-inflammatory senescent phenotype is seen in EC from veins and arteries indicating that it is a general property of ECs.

### p38 MAPK activation influences the anti-inflammatory phenotype

**Figure 9:**
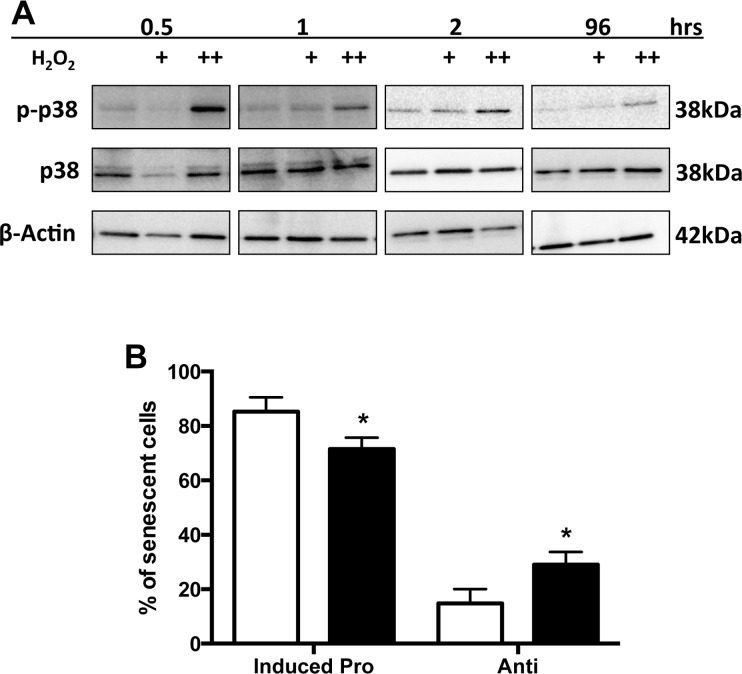
p38 MAPK activation influences the anti-inflammatory phenotype (**A**) HUVECs were treated with either 0.02 mM (+) or 0.2 mM (++) H_2_O_2_ and lysates analysed for phosphorylated p38 and total p38 levels. β-Actin was used as a loading control. This is a representative of 3 HUVEC lines. (**B**) HUVECs were untreated or treated with the p38 inhibitor SB203580 (10μM) for 2 hours. The cells are then treated with 0.2 mM H_2_O_2_ for 4 days, stimulated with TNFα and neutrophil adhesion assessed by the static adhesion assay. The number of anti and inducible pro-inflammatory senescent cells from 300 senescent cells from 6 HUVEC lines is shown. The mean percentage +/−SD is given, * p<0.05 compared to untreated (paired Student's t-test).

p38MAPK has been linked to senescence and SASP induction in fibroblasts by multiple stimuli [24, 25]. However, in contrast to that seen for irradiation induced senescent fibroblasts [26], p38 was rapidly activated in EC by H_2_O_2_ treatment (Figure 9A), with maximum stimulation seen within 30 minutes but remaining elevated even after 4 days. p38 inhibition, using the inhibitor SB203580, did not alter the overall number of senescent cells induced by H_2_O_2_, but significantly decreased the number of TNFα induced pro-inflammatory senescent cells and increased the number of anti-inflammatory senescent cells (Figure 9B). Importantly, there was no effect of the inhibitor on the ability of normal non-senescent cells to support neutrophil adhesion after TNFα, indicating that the inhibitor was not blocking the TNFα signaling pathway (data not shown).

The molecular basis of resistance to inflammatory stimuli in senescent EC to give the anti-inflammatory phenotype is not known nor is it understood the “decision” process that results in the separate inflammatory phenotypes. However, we do know that the anti-inflammatory and pro-inflammatory phenotypes do not correlate with levels of ROS, p53 or of the levels of SENEX/ARHGAP18 (data not shown).

## DISCUSSION

One of the critical hallmarks of the endothelium is the ability to support and resolve inflammatory responses. Given the prominent role of the endothelium in both inflammation and in the ageing process it is important to understand how the endothelium responds to this situation. Endothelial senescence has been connected from *in vitro, in vivo*, and most recently by *ex vivo* genomic analysis to ageing, but the functional properties of senescent EC have not been widely studied. We now show clear evidence for dynamic changes in the inflammatory phenotype of EC undergoing senescence, and importantly define an anti-inflammatory senescent phenotype, induced with a variety of stimuli that are associated with ageing.

Using neutrophil adhesion and expression of adhesion molecules, the classic markers of the inflammatory status of senescent cells, we show for the first time, that a population of senescent cells displays a powerful anti-inflammatory phenotype with an inability to support neutrophil adhesion and transmigration even after TNFα stimulation. In addition, there is a population of senescent cells that displays a pro-inflammatory phenotype, similar to that of normal EC following TNFα stimulation. Under static adhesion assay conditions the pro-inflammatory phenotype is in the majority. However, when the neutrophil adhesion assays are performed under physiological flow conditions, the anti-inflammatory population is approximately 50% of the total number of senescent cells. This is confirmed when the cells are co-stained for adhesion molecules. The anti-inflammatory phenotype is not transient, as by 6-8 days after oxidative stress stimulation the pro and anti-inflammatory populations exist in approximately equal numbers, which remain constant even out to 16 days. Thus, although we see the expected pro-inflammatory nature of senescent EC, when investigated at the cellular level, we expose the presence of another population, the anti-inflammatory population. Importantly, this mosaic of inflammatory senescent states is not just a feature of EC grown from veins, as we see these also in coronary arterial EC.

A further important finding of our study is that disturbed flow and hypoxia also induce senescence and the anti-inflammatory phenotype. Alterations in shear stress can regulate gene expression and function of EC. In *in vitro* model systems using laminar flow, EC are aligned in the direction of flow and there is an upregulation of cytoprotective genes [27]. *In vivo*, areas within arteries which are exposed to pulsatile laminar flow are protected from the development of atherosclerosis [28], whereas atherosclerosis develops at regions of bifurcation, where there are major changes in the shear stress [10] and these are the regions where EC have been identified with characteristics of senescent cells [29, 30]. Indeed, our results show that disturbed shear stress does induce senescence. We have not elucidated the mechanosensor for flow that mediates senescence induction although possibilities include primary cilia [31], integrins [32], caveolae [33] and the junctional complex of VE cadherin/PECAM/VEGFR2 [34]. Interestingly caveolae have been implicated in senescence induction [35] and we have preliminary evidence to suggest they are also involved in *SENEX/ARHGAP18* and oxidative stress-induced senescence (Powter et al manuscript in preparation). The literature regarding hypoxia and senescence is contradictory in many respects, at least in part due to the fact that hypoxic conditions and cell types vary between experiments. Many reports, including those that show hypoxia induces cell cycle arrest (but not senescence) via p53 [36], describe relatively early timepoints (≤24 hours) or modest hypoxia (≥1% oxygen). However, here we show that in EC the hypoxia needs to be both severe (≤0.5% oxygen) and sustained (5 days) before senescence becomes apparent. In tumours and inflammatory conditions, the oxygen tension can approach zero *i.e.* anoxia [37], and thus the degree and time of hypoxia used here are likely to be pathophysiologically relevant. Taken together our results show that the anti-inflammatory phenotype is not stimulant specific but is induced upon oxidative stress, shear stress and hypoxia, in addition to the overexpression of the gene, *SENEX/ARHGAP18* as we showed previously [18].

p38MAPK, a member of the mitogen activated protein kinase family, has been identified as a major regulator of senescence and indeed, in some cells overexpression of activated p38 is sufficient to induce senescence [26]. p38 activation can regulate cell cycle progression through the canonical DDR response and activation of p53 and p16 INK pathways [26, 38]. It is also involved in the development of the SASP response through NFkB activation but independent of the DDR pathway [26, 39]. In addition, the nuclear shape alterations, characteristic of senescence, are mediated through lamin B1 upregulation, in a p38 dependent manner [24]. The timing of activation of p38 to control these aspects of senescence can be different, with rapid activation leading to cell cycle inhibition whereas a delayed but more sustained activation being linked to SASP induction [26]. However, these studies have principally been performed in fibroblasts and epithelial cells and it is known that cell type and stimulant specific responses exist in the induction of senescence [40]. In EC senescence, we show activation of p38 occurs rapidly but inhibition of this activation has no effect on the senescence levels or on morphology and consistent with this, there is no change in levels of p53 and p21. Thus the growth arrest in EC is mediated through the classic DDR/ATM/p53 response but in a p38 independent manner. However, the activation of p38 does impact on the type of inflammatory profile of the senescent cell since inhibition of p38 activation switches the phenotype from the pro-inflammatory phenotype towards the anti-inflammatory phenotype. This is consistent with p38 being a pro-inflammatory signaling molecule.

The physiological ramifications of these senescent phenotypes of EC in the vasculature awaits further investigations and suitable markers for their identification. However, the results suggest that senescent EC could potentially be either protective against chronic inflammation, as in the case of the anti-inflammatory phenotype, or propagate an inflammatory state, as in the case for the pro-inflammatory senescent phenotypes. Finally, these experiments extend the concept of the antagonistic pleiotrophy of senescence [41]. The anti-inflammatory senescent cells may show benefit early on to limit, at the vascular level, disease progression. However, accumulation of these senescent cells (or lack of clearance) may contribute to the immunosenescence seen in the aged.

## MATERIALS AND METHODS

### Cell culture

Human umbilical vein endothelial cells (HUVECs)- were isolated as previously described ([18] and used between passage 1-3. Human coronary artery endothelial cells were obtained from Cell Applications (USA) and used between passage 2 and 15.

### Cell treatments

HUVECs at a confluence between 30 to 50% were treated with 0.2mM hydrogen peroxide. After 24hrs the media was changed. HUVECs at 30-50% confluence were treated with 10uM of the p38 inhibitor SB203580 (Cell Signalling). After 2hrs the media was changed.

### Senescence Associated Beta galactosidase staining

Cells are washed in PBS and then fixed in a solution containing 2% (v/v) formaldehyde and 0.2% (v/v) glutaraldehyde for 15 minutes. The cells are then washed in PBS and water and stained in a solution containing 0.22mM MgCl_2_, 0.002% NP-40, 0.001% deoxycholate, 4mM Sodium citrate, 15mM NaCl,, Potassium ferrocyanide (5mM), 100x Potassium ferricyanide(5mM), X-gal in DMF (1mg/ml) prepared in MQ water. The cells are stained for 24 hrs.

Neutrophil and mononuclear cell Isolation. Neutrophils were prepared from fresh blood from healthy volunteers. Blood was dextran sedimented, cells were separated by Histopaque (Sigma-Aldrich) gradient centrifugation and the neutrophils were purified from the cell pellet with hypotonic lysis of the remaining red cells.

### Adhesion Assay

HUVECs were plated on onto 12 well plates for 24 h. Monolayers cultured on the plates were preincubated with TNFα (5 ng/ml) for 5 h before the assay, and then washed. Neutrophils (8×10^5^/cm_2_) were added to the HUVECs. After 60 min the wells were washed and non-adherent neutrophils were removed. Photographs and counts of ECs supporting neutrophil adhesion and transmigration were made.

### Neutrophil-endothelial interactions under flow

The interaction of neutrophils with senescent endothelial cells under flow was visualized using a parallel flow chamber (Glycotech). The flow chamber was placed on a 3.5 cm diameter cell culture dish and the chamber was perfused with the warm (37.2°C neutrophil suspension (1 million cell/ml) using a syringe pump (KDScientific,). Shear force was increased gradually as described earlier [42]. Briefly; neutrophils were accumulated at a shear force of 0.25 dyn/cm^2^ followed by the exposure of shear forces of 0.5, 1, and 2 dynes/cm^2^ for 2 minutes. After 1-minute incubation period a streamline acquisition (1 frame/sec) was taken at 4 dyn/cm^2^ shear force using an Olympus F-View digital camera attached to an inverted Olympus IX71 phase contrast microscope. Images were processed using a Cell P 3.4 software (Olympus).

### Endothelial monolayer under flow

To study the relation between shear stress and senescence flow chamber slides (Ibidi m-Slide 0.4 Luer, Ibidi GmbH, Germany) were coated with 50 μg/ml fibronectin in phosphate buffered saline (PBS) overnight. HUVECs were seeded in slides at a concentration of 60.000 cells/cm^2^ to make an about 70-90% confluent monolayer. The slides were subjected to different shear forces (4, and 20 dynes/cm^2^) 2.5 hours after cell seeding. Slides grown under static conditions served as controls. To assess neutrophil-endothelial interactions in Ibidi flow chamber slides we placed 5 million neutrophils in 5 ml media in each reservoir (a total of 10 million neutrophils). Cell cultures were exposed to the shear force of 0.75 dynes/cm2 for 20 minutes (accumulation period) followed by the increase of 1 and 2 dynes/cm2 for 2-2 minutes before we switched to the target shear force (4 dynes/cm2). Neutrophils were accumulating on the surface of the endothelial layer while circulating between the two reservoirs. After 1-minute incubation period at the final shear force multiple streamline acquisitions (1 frame/sec) were acquired using a Nikon Digital Sight camera attached to a Nikon Eclipse TI Microscope. Images were processed using a Nis-Elements software (Nikon).

### Immunoprecipitations and Immunoblotting

UVECs were lysed in ice-cold lysis buffer (50 mM Tris.HCl, pH 7.4, with 1% NP-40, 150 mM NaCl, 2 mM EGTA, 1 mM NaVO_4_, 100 mM NaF, 10 mM Na_4_P_2_O_7_, and protease inhibitor cocktail (Sigma-Aldrich). Equal amounts of protein were loaded onto 10% polyacrylamide gels, separated by SDS-PAGE, transferred to polyvinylidene difluoride (PVDF) membrane, blocked with 5% skim milk powder and 0.1% Tween20 in phosphate-buffered saline (PBS), and probed with mouse monoclonal anti-beta actin (Sigma-Aldrich), monoclonal mouse anti p21^Cip1/WAF1^ (Zymed laboratories), mouse monoclonal anti-human p53 (Life Technologies), mouse monoclonal-Phospho-p38 MAPK (Thr180/Tyr182) (Cell signaling), rabbit polyclonal-p38 MAPK (Cell Signalling), mouse monoclonal anti- Anti-gamma H2A.X (phospho S139)After washing, membranes were incubated with anti-rabbit or anti-mouse secondary antibody and reactive bands were detected by chemiluminescence (ECL Western Blotting Detection Reagents; Amersham Pharmacia Biotech).

### Immunostaining

HUVECs were plated on fibronectin coated labtek slides (Invitro technologies) at 6×10^4^ for 24 h. Monolayers cultured on the slides were preincubated with TNFα (5 ng/ml) for 5 h and then washed. The cells were fixed in 4% paraformal-dehyde/PBS for 10 min, and permeabilized by treatment with 0.1% Triton X-100. Primary mouse monoclonal antibodies targeting ICAM1, E-selectin and VCAM1 were used at 20μg/ml and binding detected by incubation with Alexa 594 and 488 fluorophore-coupled secondary antibody (Life Technologies). DAPI was used as a counter stain at 400 μg/ml.

### Hypoxia induced senescence

Hypoxic conditions were achieved by placing the cells in a Mini-Galaxy A (HDScientific) hypoxic incubator (0.5% oxygen unless otherwise stated, 5% CO_2_, 37°C) in complete medium for 1-5 days. Hypoxic cells were compared to cells cultured under normoxic conditions that had not had a media change for the equivalent duration of hypoxic culture.

### Chemical activation of HIF

The prolyl hydroxylase inhibitor [21] DFO (deferoxamine mesylate; Sigma-Aldrich) was solubilised in distilled water and used at a final concentration of 10μM. Cells were treated for 72 hours to avoid toxicity problems seen after ≥5 days of culture.

### Image analysis

To analyse immunofluorescence staining we used the mean pixel intensity measurement on Image J software.

### Statistics

Statistical analyses using a paired Student's *t* test performed using Prism software (version 4; GraphPad Software, Inc.). Data that satisfy confidence levels of p < 0.05, or 0.001 are noted. Data are presented as means ± SD.

### Ethics

Human ethics was obtained from the Sydney Local Health District, Ethic Review Committee.

## 

### Videos

All videos are in a MP4 container, and have been compressed using a H264 codec. Each video was taken at 1frame/sec and reproduced as 10 frames/sec.

#### Video 1. Normal HUVECs do not support neutrophil adhesion under flow conditions is found at Video 1

HUVECs were grown on 2.5cm culture dishes. After 3 days purified neutrophils were flowed over the HUVECs at 1×10^6^/ml at a flow rate that induces neutrophil rolling (see materials and methods). Videos were taken at random locations in the dish. Video is a representative of 4 HUVEC lines. Bar=100μm.

#### Video 2. TNF stimulated normal EC support neutrophil adhesion is found at at Video 2 link

HUVECs were grown on 2.5cm culture dishes. After 3 days the cells are treated with 5ng/ml TNFα for 5hours. Purified neutrophils were flowed over the HUVECs at 1×10^6^/ml. Videos were then taken at random locations in the dish. Video is a representative of 4 HUVEC lines. Bar=100μm.

#### Video 3. Anti-inflammatory phenotype of senescent cells under flow conditions is found at at Video 3 link

HUVECs were grown on 2.5cm culture dishes and treated with 0.2 mM H_2_O_2_. After 3 days the cells are treated with 5ng/ml TNFα for 5hours. Purified neutrophils were flowed over the HUVECs at 1×10^6^/ml. Videos were then taken at random locations in the dish. The senescent cell is circled in red. Video is a representative of 4 HUVEC lines. Bar=20μm.

#### Video 4. Pro-inflammatory phenotype of senescent cells under flow conditions is found at at Video 4 link

HUVECs were grown on 2.5cm culture dishes and treated with 0.2 mM H_2_O_2_. After 3 days purified neutrophils were flowed over the HUVECs at 1×10^6^/ml. Videos were then taken at random locations in the dish. The senescent cell is circled in red. Video is a representative of 4 HUVEC lines. Bar=20μm.

#### Video 5. Shear stress induced senescent cells show the anti-inflammatory phenotype is found at at Video 5 link

HUVECs were plated onto Ibidi slides and were subjected to 24hrs of flow at 4 dyne/cm2. The cells were then treated with 5ng/ml of TNFα for 5 hours. Purified neutrophils were then flowed across the surface of the HUVECs at a shear force that allows neutrophil rolling and adhesion. The senescent cell is circled in red. This is a representative of 3 HUVEC lines. Bar=100μm.

#### Video 6. Shear stress induced senescent cells show the inducible pro-inflammatory phenotype is found at at Video 6 link

HUVECs were plated onto Ibidi slides and subjected to 24hrs of flow at 4 dyne/cm2. The cells were then treated with 5ng/ml of TNFα for 5 hours. Purified neutrophils were then flowed across the surface of the HUVECs at a shear force that allows neutrophil rolling and adhesion (See materials and Methods). The senescent cell is circled in red. This is a representative of 3 HUVEC lines. Bar=100μm.

## SUPPLEMENTARY VIDEOS












